# “From That Moment, Everything has Changed”: The Experience of Women With Anorexia Nervosa Receiving a Diagnosis of Autism

**DOI:** 10.1002/erv.3186

**Published:** 2025-03-09

**Authors:** Melissa Creese, Sarah Hampton, Janina Brede, Charli Babb, Mair Elliott, Lucy Serpell, Catherine R. G. Jones, John R. E. Fox, Alana Loewenberger, Will Mandy

**Affiliations:** ^1^ Research Department of Clinical Educational and Health Psychology University College London London UK; ^2^ Royal Free NHS Foundation Trust London UK; ^3^ School of Psychology Cardiff University Cardiff UK; ^4^ Haverfordwest Wales UK; ^5^ Eating Disorder Service North East London NHS Foundation Trust London UK; ^6^ School of Psychology University of Sheffield Sheffield UK

**Keywords:** adulthood, anorexia nervosa, qualitative research

## Abstract

**Objective:**

Autism and eating disorders (ED) frequently co‐occur, particularly in women. Autistic individuals are often undiagnosed when they present to mental health services and many receive their autism diagnosis during or after ED treatment. This study sought to understand the experiences of autistic women with co‐occurring anorexia nervosa (AN) receiving an autism diagnosis.

**Method:**

Secondary data analysis was conducted on 17 semi‐structured interviews with autistic women with AN using reflexive thematic analysis. Participants had a diagnosis of autism, had current or past experience of AN, were female‐identifying and aged 18 or above.

**Results:**

Participants experienced missed opportunities for autism diagnosis along with misdiagnoses and misunderstandings from healthcare professionals. Participants tended to receive their diagnosis at the point of crisis and experienced being passed between autism and ED services. Receiving a diagnosis helped participants make sense of their experiences and take control of their lives but also brought feelings of shock and distress.

**Conclusions:**

While autism diagnosis is often a positive experience for autistic women with AN, a range of emotions can be experienced. The findings highlight a need for better and earlier identification of autism among women with EDs, alongside appropriate post‐diagnosis support and ED treatment that is adapted to autistic individuals' needs.

**Summary**

- Autistic women with Anorexia Nervosa (AN) often do not receive an autism diagnosis until their mental health has reached a crisis point.
- Autistic women with AN report being passed between autism services and eating disorder services, leaving them feeling unsupported.
- Receiving an autism diagnosis can be a positive experience for women with AN, supporting both self‐understanding and self‐advocacy, though it can also bring feelings of ambivalence.

## 1 Introduction

Autism is a neurodevelopmental condition characterised by differences in social communication and interaction, sensory sensitivities, repetitive behaviours and intense interests (American Psychiatric Association 2013). Autism is a lifelong condition with characteristics emerging in early childhood, although these characteristics are not always recognised until adulthood (Bargiela et al. 2016; Lai and Baron‐Cohen 2015). In the UK, autism is typically diagnosed within autism specialist services via multi‐disciplinary team assessment based on diagnostic tools, observation, and informant report, though there is much variation in these processes (Beresford et al. 2020; National Institute for Health and Care Excellence 2021). Rates of autism diagnosis vary according to sex/gender, with the male‐female ratio of diagnosis estimated at 3:1 (Loomes et al. 2017). Women tend to receive a diagnosis of autism on average several years later than men (Atherton et al. 2021), with some autistic women reporting that it was not until experiencing a mental health crisis that their autism was identified (Harmens et al. 2022). These delays in diagnosis may in part be due to women being more likely to mask their autistic characteristics (Bargiela et al. 2016; Cook et al. 2024). Furthermore, the diagnostic criteria for autism tend to reflect male presentations of autism, potentially leading to an under‐recognition of autism in women who may be misdiagnosed with conditions such as borderline personality disorder (Dell’Osso and Carpita 2023). Autistic women are more likely to experience symptoms of mental health conditions such as anxiety and eating disorders (ED) than autistic men (Sedgewick et al. 2021). This may result in diagnostic overshadowing whereby professionals misattribute an individual's difficulties to co‐occurring mental health conditions (Babb et al. 2021; Lai and Baron‐Cohen 2015). Misdiagnosis and diagnostic overshadowing risk incorrect treatment being offered for autistic women's difficulties (Harmens et al. 2022).

Delays in autism diagnosis may have a significant impact on wellbeing (Mandy et al. 2022). Late‐diagnosed autistic women often describe the benefits of a diagnosis, which can provide them with an explanation for their differences (Kelly et al. 2022; Harmens et al. 2022). An autism diagnosis can also enable opportunities to connect with other autistic women (Kelly et al. 2022), with connectedness to autistic community and a positive sense of autistic identity being associated with improved wellbeing (Davies, Cooper, et al. 2024). An autism diagnosis can enable appropriate tailored support from health and social care professionals (Lai and Baron‐Cohen 2015). However, diagnosis does not always translate to better support for autistic women, with many describing inadequate post‐diagnostic support (Crane et al. 2018). Autistic women report a lack of knowledge of autism in women among healthcare professionals, who are not always able to recognise their challenges (Kelly et al. 2022).

While autism and mental health conditions such as ED often co‐occur in women, and late diagnosis can have an impact on the mental health of autistic women, little is known about the experience of late‐diagnosed autistic women who have accessed treatment for their mental health before receiving an autism diagnosis. It is estimated that 20%–35% of women with ED (with most studies focussing on AN) meet the diagnostic criteria for autism (Carpita et al. 2022; Westwood and Tchanturia 2017; Westwood et al. 2017) and a recent review indicates that autistic women show high levels of disordered eating behaviours (Schröder et al. 2022). Autistic people receive their autism diagnosis on average 6.1 years after their first ED diagnosis (Zhang et al. 2022), indicating that autistic women are often undiagnosed at the time of presenting to ED services. Patients with EDs and co‐occurring autism experience longer and more intensive ED treatment (Li et al. 2022), more frequent inpatient stays (Zhang et al. 2022) and longer duration of hospitalisation (Kemp et al. 2023). Autistic women also access a broader range of healthcare services and ED treatments than non‐autistic women and experience their care as less beneficial (Babb et al. 2022).

It is worth noting that research in this area often does not distinguish between autistic participants who already had an autism diagnosis at the time of accessing ED services and those who did not, making it difficult to draw conclusions about the role of an autism diagnosis in experience of ED treatment. Nevertheless, given the poorer ED‐related outcomes autistic women face, timely autism identification is crucial so that appropriate ED support can be given. One qualitative study indicated that for autistic women with ED, receiving an autism diagnosis can help them to understand how to distinguish between autistic and ED behaviours and therefore gain a better understanding of their needs (Kinnaird et al. 2018). Autistic women in this study also reported barriers to receiving appropriate ED support, such as clinicians' lack of understanding of autism, being refused treatment by ED services, and a lack of autism‐related adaptations to ED treatment (such as sensory difficulties around food and rigid thought patterns not being taken into account within treatment programs). A recent review also indicated that there is little evidence that treatment outcomes in terms of ED symptoms differ between autistic and non‐autistic people (Nimbley et al. 2024), and qualitative work suggests autistic and non‐autistic people define ED recovery in similar terms (including weight restoration, reduced ED behaviours, reduced disordered thinking and increased positive emotions; Sedgewick et al. 2022). However, there is some evidence these outcomes may be experienced differently between the two groups, with food, weight, and body images disturbances playing less of a role in the experience of and recovery from ED for autistic people (Nimbley et al. 2024).

The interaction between autism and ED is complex. The effects of starvation on the brain may mimic or exacerbate autistic traits (Keys et al. 1950; Hiller and Pellicano 2013), potentially leading to incorrect identification of autism among those with ED. Furthermore, autism and ED symptoms can have a reinforcing and maintaining effect on each other (Brede et al. 2020; Kinnaird et al. 2018). Given this multifaceted relationship, autistic women with ED may experience their autism diagnosis differently to autistic women without co‐occurring ED.

There is little data on how autistic women with co‐occurring ED experience receiving an autism diagnosis. This study aimed to explore the journey to an autism diagnosis for women with co‐occurring anorexia nervosa.

## 2 Method

### 2.1 Study Design

The present study is a secondary data analysis of semi‐structured qualitative interview data. For the original data collection, the study design and interview schedules were developed in consultation with two autistic women with experience of AN. Interview schedules covered: (1) autism and autism diagnosis; (2) AN; and (3) ED services. The present study takes the first topic as its focus and topics two and three are reported elsewhere (self‐citation). The interview schedule can be found elsewhere (Brede et al. 2020; Babb et al. 2021). Interviews were conducted in person or via video/phone call. Interviews lasted between 43 and 120 min and were recorded and transcribed verbatim.

### 2.2 Participants

Seventeen women took part. Participants were recruited using social media and the Autistica network. The inclusion criteria were: (1) above the age of 18; (2) self‐reported formal clinical diagnosis of autism; (3) current or past experience of AN; (4) identify as female; and (5) living in the UK.

### 2.3 Data Analysis

Data were analysed using reflexive thematic analysis (TA; Braun and Clarke 2021). This approach allowed for flexibility, as there was no particular theory guiding data analysis. A reflexive journal was kept by [MC] throughout the process of analysis. After a process of familiarisation (reading all transcripts twice), transcripts were inductively coded line‐by‐line by [MC], after which [AL] checked two of the transcripts to ensure the codes captured participants' meanings [MC] then combined the first‐level codes into themes. Candidate themes were reviewed against codes both within each interview and across the dataset in regular discussion with [AL]. There were a few cases where themes appeared inconsistent with the data or lacked depth, and so re‐organisation of themes took place before a final thematic structure was reached, in consultation with members of the wider team.

### 2.4 Positionality Statement

The first author is a non‐autistic woman which may mean that she missed some of the meaning behind autistic participants' words when analysing the data. She does not have experience of living with ED, although has worked clinically in these settings. While being a woman may provide first‐hand understanding of womanhood, the author may have projected some of her own experiences onto the data. The broader team included both autistic and non‐autistic people and takes a neuro‐affirmative stance to research.

## 3 Results

### 3.1 Demographics

Participants' AN status varied at the time of the interview with some still living with AN while others considered themselves recovered. All had been in contact with ED and other mental health services prior to receiving their autism diagnosis. On average the women were diagnosed with autism 11.5 years after their diagnosis of AN. Eleven participants were in full‐time employment, held part‐time jobs or voluntary posts and one was retired. Four were studying at university, and one had interrupted their studies due to the impact of AN. Demographics are presented in Table 1.

**TABLE 1:** Demographic information of participants (*N* = 17).

Demographics	Mean (SD)	Range
Age (years)	32.6 (10.3)	23–58
Age at AN diagnosis (years)	17.9 (6.0)	11–34
Age at autism diagnosis (years)	29.4 (11.3)	14–56
Current BMI (*n* = 7)^a^	18.5 (3.1)	15.3–23.1

*Note:* AN, Anorexia Nervosa; BMI, Body Mass Index; SD, Standard Deviation.

^a^ Data is missing for 10 participants as not all participants were willing to provide their height and weight. There were no missing data for any other variables.

## 4 Themes

Three themes and nine subthemes were developed (Table 2). Participants are identified by anonymised codes.

**TABLE 2:** The themes and sub‐themes identified through reflexive thematic analysis.

Themes	Sub‐themes
1. The search for understanding and support	1.1 Searching for a place to fit
	1.2 Mis(sed) diagnosis and misunderstanding
	1.3 Journey to crisis point
	1.4 Passed from service‐to‐service post diagnosis
2. A shifting moment	2.1 Everything makes sense
	2.2 Shock and upset
	2.3 What differences does diagnosis make?
3. Taking control	3.1 A communication tool
	3.2 Forging my own path post‐diagnosis

### 4.1 Theme 1: The Search for Understanding and Support

This theme describes participants' experiences of searching for understanding of themselves and support for their struggles, both prior to and after autism diagnosis.

#### 4.1.1 Searching for a Place to Fit

Participants spoke about experiences of feeling different and othered by society, bringing about feelings of loneliness, ‘*I've always felt like I'm on the outside looking in’ (Autistic Woman [AW] 11).* Receiving the diagnosis of autism felt for some like they finally had a community where they belonged, enabling them to be themselves.

AW08: …it also made me feel less alone because then I realised that there are other people who also have both [ED and autism], and other people have similar experiences.

However, for others, receiving an autism diagnosis did not open up opportunities to fit in. They described not feeling supported by their friends and family post diagnosis, and the search for a place to be understood continued.

AW11: I don’t think people reacted in a good way particularly… I think people misbelieve me, they see me as so functional and they see how much I'm able to achieve, but they don’t see how much pedalling is going on beneath the surface…

#### 4.1.2 Mis(sed) Diagnosis and Misunderstanding

While for most participants autism had been suspected previously, particularly at school, they were not formally diagnosed until adulthood, usually after presenting to ED services.

AW01: I was under the [ED] services in England since I was 14, I am 25. And I have never had a proper psychiatric assessment outside of an ED setting. So [GP] suggested I do that.

Experiences of misdiagnosis were common, ‘*they previously diagnosed me with borderline personality disorder but I didn't relate to it at all’* (AW16). Some reported receiving not just one prior diagnostic label but several that they felt were misdiagnoses.

AW13: I mean I did get a diagnosis aged 16 of OCD and depression and then the diagnoses just kept coming. Social anxiety blah blah blah. So, I seemed to collect a whole set of mental health diagnoses…

This highlights the misunderstandings services may have of the autistic experience in individuals with co‐occurring mental health difficulties, which some participants felt were exacerbated by a lack of understanding about the female autistic experience, ‘*They didn*'*t seem [to be] a very clear definition of the boundaries of the [autism] diagnostic criteria they applied to females’ (AW10).*

The experiences of misdiagnoses and late diagnosis led some to reflect on what an earlier diagnosis could have given them. For some, this was with a lens of compassion for their past selves, but for others this brought frustration about the difference an earlier diagnosis could have made to their lives.

AW08: If only I’d known different, I could have possibly managed this with some adaptions, I could have possibly done that, I possibly wouldn’t have been so hard on myself on achieving things.

#### 4.1.3 Journey to Crisis Point

Not only did most participants receive their diagnosis of autism later in life, but the diagnosis tended to come at the point of breakdown and burnout. It seemed as though only at this point of breakdown did mental health teams consider additional explanations such as autism.

AW03: I think I had years of struggling with my mental health and anorexia and I reached a point where I was just, I was completely burned out, and I had, I suppose a breakdown. After that point I had to stop work. …They spent about half a day with me and at the end of the assessment they suggested I had ASD.

This meant participants were left searching for an understanding of their struggles and why the ED treatment they were offered was not effective in resolving their difficulties.

AW15:…what’s wrong with me because I'm just going mad. I can't fit in, I can't get on with people, I can't hold down a job, so if it’s not what everybody’s saying it is [AN] or what it could be, what on earth is it? I was starting to think I had a brain tumour and all sorts of things because I couldn’t cope.

#### 4.1.4 Passed From Service‐To‐Service Post Diagnosis

Even when the autism diagnosis was made, participants described a continued search for understanding and support from ED and autism services. Participants spoke about being offered either treatment with an ED service *or* support from an autism service, but rarely both. They often experienced being passed from service to service, with some being refused treatment.

AW05: I think there wasn’t a kind of, the services don’t seem to meet…. So you have an Asperger’s service, an autism service around if you’re lucky, an ED service around if you’re lucky. But… There doesn’t seem to be a kind of merging of them.

This left participants with a feeling of not being listened to and not fitting in, which echoed a prominent feature of their lives to date.

AW11: I go to the autism service, and even going there I find really difficult. I feel like I don’t even fit in with – I can notice that we have similarities but at the same time I still don’t know how to connect with people very well.

The participants also described a significant lack of support after their autism diagnosis. Services tended not to offer ongoing post‐diagnostic support, meaning many were not given opportunities to process their diagnosis and its possible implications (such as adaptations and decisions around disclosure). Some participants did describe helpful support after their autism diagnosis, particularly when support addressed both autism and AN. However, this was rarely offered to them by NHS services.

AW03: Then I found out about a charity that provides autism specialist counselling. I contacted them, and I had that…and that has been incredible. Because I feel, we work on the autism, but we also work on the ED as well, so it is like a more holistic, I suppose, and that has been really effective.

### 4.2 Theme 2: A Shifting Moment

This theme captures how receiving an autism diagnosis affected participants, including the emotional impact at the point of diagnosis and how diagnosis became a shifting moment in the participant's narrative.

#### 4.2.1 Everything Made Sense

Diagnosis for some was a light‐bulb moment, shedding light on why they had struggled in life and providing relief, ‘*from that moment everything has changed’ (AW03).* Participants felt that a diagnosis of AN did not fully explain their difficulties and autism provided a useful explanatory framework for their mental health struggles and other differences.

AW03: There were a lot of differences that weren’t explained by the anorexia. And we would try constantly “there is something else, there is something else”. And after all these years, I finally found what this something else was.

Participants were able to make links between autism and their AN, beginning to hypothesise about why and how their AN may have occurred.

AW17:…knowing about autism, knowing about mental health in autism, I now understand that all these things came on the foundation of the autism.

Being able to give a name to their struggles subsequently became a shift in their narrative for some participants, enabling a better understanding of themselves. This brought a feeling of self‐acceptance.

AW12: I've always felt different and like I didn’t fit in and it was kind of like oh actually that’s because I am different and I don’t particularly fit in and that’s okay. And then I could kind of see ways of working with it as a strength rather than letting it hold me back.

#### 4.2.2 Shock and Upset

For some participants, feelings of shock and upset were described when receiving their autism diagnosis. This was often in the weeks following diagnosis and, when combined with feelings of relief and self‐acceptance described above, left many feeling confused. For some, the shock and upset related to a realisation that autism is a lifelong condition.

AW01: [Autism is] not something you get rid of, while an eating disorder, people gave me the impression that if I recover from an eating disorder, my life would be, you know, the difficulties would all go away.

For some participants, the shock and upset felt after receiving the autism diagnosis was in part due to their families' reactions, who found it challenging to understand their diagnosis and sometimes experienced guilt.

AW01: I think my parents were quite, especially my mom, a bit guilty….just because, maybe because, she didn’t push even more to investigate I guess. Especially when I was younger…

This left some participants needing time after their diagnosis to process their diagnosis. Participants described a range of reactions to their diagnosis, with some even rejecting the diagnosis.

AW10: You know, I don’t feel I can objectively say whether or not I am autistic, I really don’t, because I’ve never really understood what it should look like.

#### 4.2.3 What Difference Does Diagnosis Make?

While the feeling that an autism diagnosis made sense was widespread, it was not universal. For a small group, receiving the diagnosis brought indifference and ambivalence. This ambivalence was reflected in being unsure how autism affects them, or not being supported to develop a full understanding of autism and its relevance to their experience.

AW10: I didn’t really feel it was me, but then again I didn’t really feel I had enough information to make an informed judgement… I’m still quite ambivalent, yes.

Some questioned what difference a diagnosis actually made, as the difficulties they faced did not improve post‐diagnosis as much as they had hoped. This was especially true for those receiving a diagnosis later in life, as many participants had already developed strategies to navigate life by the time of diagnosis.

AW08: But I think for me and possibly for others it is a slightly different ball game, when it’s been undiagnosed for decades and I’ve had to function, which I have.

### 4.3 Theme 3: Taking Control

This theme details feeling able to take control after receiving an autism diagnosis and using the diagnosis as a tool to forge a new path in life.

#### 4.3.1 A Communication Tool

Many participants felt that receiving a diagnosis gave them a tool to explain their differences, help others understand them, and advocate for their needs. Participants could explain to others why certain adjustments were needed, which enabled them to cope better. Knowing they were autistic gave them a way to express their emotional struggles to others in words rather than these struggles manifesting in other ways.

AW03: With this information I was then able to explore all the difficulties. I had the words to communicate, whereas before my way of communication was very much by not eating.

Some participants felt that receiving an autism diagnosis enabled their ED service to better understand their needs. Good examples of integrated care across services were described following diagnosis, where autism services were able to help plan AN treatment. Positive experiences of psychological therapy post‐diagnosis were also described, often because knowledge of the participant's autism enabled therapists to adapt treatment and consider autism within their formulation.

AW11: That was kind of a bit of a turning point in that [therapist] understood like how best to communicate with me so she gave me written summaries after my appointments, she gave me written information, we used kind of goal setting to help plan my care.

#### 4.3.2 Forging my Own Path Post‐Diagnosis

The majority of the participants described how an autism diagnosis enabled them to make beneficial adaptions to their lives. Some described adaptations to ED care.

AW03: So [the inpatient unit] created a different space that was separate, because I think they realised for my stay there, it wasn’t appropriate for me to go through the whole treatment programme.

Participants described taking control of their lives by making their own adaptions, creating a life that was accommodating of their autism. Many participants viewed autism as a strength and described how this could bring self‐compassion, ‘*I can be a bit more compassionate to myself because it's not my fault that I find certain things difficult.’ (AW13*).

Some participants discussed that having a diagnostic label meant they could do their own research into autism and find support groups, which subsequently helped them learn how to manage better. While this reflected the fact that most were given limited post‐diagnostic support and information from services, learning about autism also represented a way of forging a path to understanding themselves. Receiving an autism diagnosis therefore meant for some that they could move forward with their lives, armed with new self‐knowledge, ‘*It wasn't closure, it was almost like that was the start for the next chapter of my life.’ (AW03*).

## 5 Discussion

The present study is the first to specifically explore the journey to autism diagnosis among women with a prior diagnosis of AN. Participants discussed a continued search for understanding and support, both prior to and after receiving their autism diagnosis. They reported missed opportunities for diagnosis earlier in their lives and misdiagnoses along the way. Autism diagnosis generally occurred at the point of crisis and resulted in being passed between ED and autism services. The women commonly experienced receiving their diagnosis as a shifting moment in their life, with some feeling that autism helped explain their continued struggles, while others felt shocked and upset upon receiving the diagnosis. For others, there was no shifting moment and they were instead left questioning how this new information would make a difference to them. Taking control was also a key theme and participants described how the diagnosis became a communication tool to explain their needs and make adaptions, allowing them to forge their own path in life post‐diagnosis.

The present study replicated previous findings that autistic women experience multiple misdiagnoses and missed opportunities for diagnoses earlier in life (De Broize et al. 2022; Leedham et al. 2020). Women were often diagnosed at a later stage of life and regretted lacking access to diagnosis and support earlier. Our findings corroborate quantitative studies suggesting that individuals with EDs often receive an autism diagnosis many years after their ED diagnosis (Zhang et al. 2022; Brede et al. 2024). In addition, this study highlights that diagnosis tended to occur at the point of mental health crisis, after long, and often unsuccessful, treatment for AN. This phenomenon does not appear to be unique to autistic women with co‐occurring AN. Harmens et al. (2022) found that reaching a breaking point also led to identification of autism for autistic women with other co‐occurring mental health diagnoses, such as depression and PTSD. This may highlight the role of diagnostic overshadowing in the autism diagnostic journey (Lai and Baron‐Cohen 2015), with professionals assuming that autism characteristics are the result of mental health struggles (Babb et al. 2021). It is therefore important that services consider a neurodevelopmental picture alongside mental health diagnoses from the first presentation to services to reduce the likelihood of autistic women missing out on autism‐informed care and reaching breaking point.

Misdiagnosis and delayed diagnosis for autistic women with AN may in part be due to the lack of understanding mental health professionals have of the female experience of autism. A recent systematic review found that healthcare professionals report only moderate levels of autism knowledge and self‐efficacy in working with autistic individuals, and often lack training in this area (Corden et al. 2022). As healthcare professionals play a key role in identifying and supporting autistic individuals, more training is needed to identify autism earlier. Recently, the Health and Care Act (2022) introduced specific requirements that English NHS services must ensure their staff receive learning disability and autism training. This led to the creation of The Oliver McGowan Mandatory Training on Learning Disability and Autism (Health Education England 2022), which is mandatory for all health and social care staff. Specific training initiatives such as this may help support professionals to identify autism earlier and reduce health inequalities, enabling autistic people to receive the support they need to flourish.

Participants in the present study often found receiving an autism diagnosis beneficial. It enabled them greater self‐knowledge and compassion and to advocate for their needs to friends, family and support services, echoing previous findings among autistic women without co‐occurring mental health diagnoses (Kelly et al. 2022; Crane et al. 2018; Harmens et al. 2022). Diagnosis also allowed them to find community among autistic people. This was especially important due to feeling different to other patients with AN, which perpetuated the experience of being othered they had felt throughout their lives.

Prior literature indicates the importance of appropriate support to enable integration of autism into one's sense of self in a positive, strengths‐based way (Cooper et al. 2023; Kelly et al. 2022). This could include use of the affirmative model suggested by Swain and French (2000), which aims to embrace positive social identities and diverse ways of being. National Institute for Health and Care Excellence (NICE) guidance also sets out that adults who receive a diagnosis of autism should be offered psychoeducation to discuss implications of the diagnosis and future support, as well as psychosocial interventions and interventions for co‐existing mental health difficulties where needed (NICE, 2021). However, how often this happens within services and whether this is sufficient to process the diagnosis is uncertain. Autistic women in the present study sometimes reported feeling unsupported and abandoned post‐diagnosis. Navigating the gap between autism diagnosis and support requires significant knowledge, time, finances and personal resources (Huang et al. 2022) and autistic adults report wanting more information, advice and professional assistance to find support after diagnosis (Crane et al. 2018). Appropriate post‐diagnosis support and information is therefore crucial for autistic women to adjust, adapt and process what autism means to them while also finding community. This may alleviate the feeling of diagnosis being a burden and negatively affecting their mental health.

It is also possible that internalised stigma of autism was experienced by some participants. Many reported feelings of shock and upset, not knowing how to discuss diagnosis with their friends and family, and difficulty processing that autism is a life‐long condition. Autistic individuals experience frequent exposure to stigma attached to autism by society (Botha et al. 2022). Experiences of internalised stigma are common and have been shown to be more severe in autistic individuals aged over 35 years (Bachmann et al. 2019) indicating that late‐diagnosed autistic individuals may be at higher risk of developing internalised stigma. Further research should therefore focus on the extent to which autism‐related stigma underlies the different emotional reactions to autism diagnosis, as this could be an important focus for post‐diagnostic support. Given that a positive sense of autistic identity and access to external support are linked to greater wellbeing (Davies, Cooper, et al. 2024), the provision of peer support to foster positive identity development for autistic women with AN may be beneficial (Davies, Redmayne, et al. 2024).

Specific post‐diagnostic support for autistic women with AN appears to be limited and participants often felt that professionals lacked understanding of the presentation of autism in women and the interaction between autism and AN. The interaction between autism and ED is complex, with features such as restrictive eating, cognitive rigidity and difficulties in social and emotional functioning being common to both conditions (Kinnaird and Tchanturia 2021; Kinnaird et al. 2018). It may be that clinicians do not feel equipped to navigate these complexities. Indeed, recent qualitative work suggests clinicians lack confidence treating those with co‐occurring autism and ED and receive little systematic guidance on how to adapt ED treatment for autistic people (Kinnaird et al. 2017). Multiple participants reported being passed between autism services and ED services, ultimately leaving them unsupported at a crucial time in their lives. The Pathway for EDs and Autism developed from Clinical Experience (PEACE pathway) provides treatment resources and advice for clinicians and explains what tailored, autism‐adapted ED support should look like (Tchanturia et al. 2020). The pathway emphasises the importance of ED services providing appropriate support for autistic people, for example, by addressing possible sensory sensitivities, reducing use of open‐ended questions, and offering individual instead of group therapy (Tchanturia et al. 2020). Recent qualitative research with clinicians indicates that the PEACE pathway brings benefits such as increased clinician confidence and understanding when working with autistic people with ED, alongside challenges such as incorporating adaptations into existing ED treatment protocols (Li et al. 2024). A recent review also suggests that the programme may lead to reduced length of hospital admissions and reduced treatment costs (Li et al. 2022). Autistic women in the present study found it helpful when both autism and AN were considered within a formulation and treatment. Further work is needed to make autism‐adapted ED treatments more widely available and to test their effectiveness.

## 6 Limitations

The authors leading the analysis did not collect the data and the original dataset was mainly intended to answer different questions than those explored here (Babb et al. 2021; Brede et al. 2020). This may have led to fewer follow‐up questions on the topic of receiving an autism diagnosis and some information may have been missed. The present study sought to explore the experiences of autistic women with co‐occurring AN. Due to the complex and specific interplay between *restrictive* EDs and autism, it is possible that the findings of this study only represent experiences of autism diagnosis and co‐occurring AN rather than other co‐occurring EDs. Furthermore, as the effects of AN‐related starvation on the brain may mimic autistic traits (Hiller and Pellicano 2013), it is possible that some participants may have received an autism diagnosis in error.

The present research was conducted only with women and it is possible that autistic men and gender diverse individuals could experience autism diagnosis differently. Indeed, gender diverse individuals are more likely to be diagnosed as autistic later in life (which could have implications for their vulnerability to developing mental health difficulties; Hisle‐Gorman et al. 2019). It is possible there was selection bias in the sample; the present study does not represent the experience of those who did not or could not volunteer to participate, including those without the verbal ability to take part in an interview, although adaptations were offered to make participation more accessible for example providing questions in advance, and using written communication as part of the interview process. Because of the recruitment methods used, the sample is biased toward those who were already linked with Autistica's networks or who used social media. The views of autistic women not actively seeking research participation, or not motivated to take part in research, are likely underrepresented. Another limitation is that participants were not required to provide official documentation of their autism and AN diagnoses. However, participants gave consistent details of their diagnostic experiences during the interviews, giving researchers no reason to doubt the authenticity of their diagnoses.

## 7 Conclusion

The present study highlights that receiving an autism diagnosis is an important moment in the lives of autistic women with co‐occurring AN. Our findings suggest that autism should be identified as early as possible into treatment for co‐occurring AN. This can allow for tailored support to be offered and can reduce the likelihood of misdiagnosis. Autism diagnosis can allow autistic women to better understand themselves. Autism diagnosis provides empowering information to enable individuals to find community and informal support, but also to advocate for their needs within clinical services. Further work is needed to ensure that healthcare professionals have a good understanding of autism in women, and where possible treatment for AN should be continued within their existing ED services working together with specialist autism services.

## Ethics Statement

Ethical approval was obtained from University College London (UCL) and Cardiff University for the original data collection (reference numbers: 12,973/001 and EC.18.05.08.5302).

## Consent

Participants in the original study gave written informed consent to participate.

## Conflicts of Interest

The authors declare no conflicts of interest.

## Data Availability

The data that support the findings of this study are available on request from the corresponding author. The data are not publicly available due to privacy or ethical restrictions.
